# Properties of Plant Virus Protein Encoded by the 5′-Proximal Gene of Tetra-Cistron Movement Block

**DOI:** 10.3390/ijms241814144

**Published:** 2023-09-15

**Authors:** Denis A. Chergintsev, Anna D. Solovieva, Anastasia K. Atabekova, Alexander A. Lezzhov, Sergei A. Golyshev, Sergey Y. Morozov, Andrey G. Solovyev

**Affiliations:** 1 A. N. Belozersky Institute of Physico-Chemical Biology, Moscow State University, 119992 Moscow, Russia; ledumpalustre86@gmail.com (D.A.C.); asya_atabekova@mail.ru (A.K.A.); lezzhov-genetic@mail.ru (A.A.L.); sergei.a.golyshev@gmail.com (S.A.G.); morozov@genebee.msu.ru (S.Y.M.); 2 Department of Virology, Biological Faculty, Moscow State University, 119234 Moscow, Russia; l_anna2000@mail.ru

**Keywords:** plant virus, virus cell-to-cell movement, virus movement protein, RNA silencing, viral suppressor of RNA silencing, RNA-binding protein, double-stranded RNA binding

## Abstract

To move from cell to cell through plasmodesmata, many plant viruses require the concerted action of two or more movement proteins (MPs) encoded by transport gene modules of virus genomes. A tetra-cistron movement block (TCMB) is a newly discovered transport module comprising four genes. TCMB encodes three proteins, which are similar to MPs of the transport module known as the “triple gene block”, and a protein unrelated to known viral MPs and containing a double-stranded RNA (dsRNA)-binding domain similar to that found in a family of cell proteins, including AtDRB4 and AtHYL1. Here, the latter TCMB protein, named vDRB for virus dsRNA-binding protein, is shown to bind both dsRNA and single-stranded RNA in vitro. In a turnip crinkle virus-based assay, vDRB exhibits the properties of a viral suppressor of RNA silencing (VSR). In the context of potato virus X infection, vDRB significantly decreases the number and size of “dark green islands”, regions of local antiviral silencing, supporting the VSR function of vDRB. Nevertheless, vDRB does not exhibit the VSR properties in non-viral transient expression assays. Taken together, the data presented here indicate that vDRB is an RNA-binding protein exhibiting VSR functions in the context of viral infection.

## 1. Introduction

Plant viruses are transported from infected cells to surrounding healthy cells through plasmodesmata (PD), the channels that connect cells anddetermine therefore the symplastic nature of plant tissues [1,2]. It has long been known that viral transport through the PD channels cannot occur by passive diffusion but, rather, is an active process that requires virus-encoded polypeptides called movement proteins (MPs) [3].

Replication of viruses with single-stranded RNA genomes of positive polarity, which constitute the majority of plant viruses, occurs in the cytoplasm in association with cell endomembranes [4]. Current evidence suggests that virus replication and cell-to-cell transport are closely linked and often occur at membrane structures located in proximity to PD, and that virus-encoded MPs are involved in the reorganization of cell membranes, leading to the formation of such membrane structures [5]. MP properties have been well studied for three MPs, named TGB1, TGB2 and TGB3, which are encoded by the “triple gene block” (TGB), an evolutionarily conserved gene module consisting of partially overlapping genes found in a number of virus groups [6]. TGB1 is a helicase domain-containing RNA-binding protein that is thought to interact with viral genomic RNA or virions to give rise to the form of the viral genome destined for cell-to-cell transport [6]. TGB2 and TGB3 are small proteins with hydrophobic sequence regions that are integrated into the membranes of the endoplasmic reticulum (ER); together, TGB2 and TGB3 target TGB1 and, presumably, TGB1-containing complexes with genomic RNA to PD and through the PD channels [6]. The interaction of the small TGB proteins with the ER membranes leads to the formation of specialized ER-derived membrane bodies consisting of modified ER tubules at the PD orifice [7]. Viral replicase can be recruited to these structures through its interaction with TGB2 [8]. Thus, membrane compartments for virus replication are associated with PD, coupling virus replication and transport, as newly synthesized progeny genomic RNA can be delivered directly from the replicative compartments to the PD channels [7]. The TGB1 protein, at least in viruses of the genus *Potexvirus*, has an additional function as a viral suppressor of RNA silencing (VSR) [9,10]. Some TGB1 mutants that are deficient in both cell-to-cell transport and VSR function can be complemented by heterologous VSRs that restore the protein function in virus movement [9], suggesting that the suppression and movement functions of TGB1 can be separated and that the VSR function is required for virus cell-to-cell transport [9].

Analyses of recently discovered viral genomes and transcriptomic data have led to the identification of novel TGB-related gene modules. In particular, the genomes of viruses of the genus *Higrevirus* (family *Kitaviridae*) contain a “binary movement block” (BMB) consisting of two genes encoding the BMB1 protein, which is distantly related to TGB1, and the BMB2 protein, which shows a marginal but detectable similarity to TGB2 [11]. The BMB2 protein interacts with ER membranes and, exhibiting reticulon-like properties, generates additional membrane curvature, thereby inducing the formation of modified ER tubules that constitute ER-derived membrane bodies associated with PD [12]. Similar to small TGB proteins, BMB2 directs BMB1 to PD-associated membrane structures and through PD to neighboring cells [13]. Thus, BMB2 appears to be functionally equivalent to two TGB proteins, TGB2 and TGB3. From an evolutionary point of view, BMB can be considered as a progenitor of TGB, which could have evolved from BMB by acquiring a 3′-proximal additional third gene that could further take over some BMB2 functions [14].

Virus-like sequences are often found in plant transcriptomes, as plant tissue samples collected for new-generation sequencing may have been obtained from virus-infected plants [15]. Another TGB-related gene module, the “tetra-cistron movement block” (TCMB), has recently been discovered in the transcriptomic contigs of the moss *Dicranum scoparium* and the flowering plant *Colobanthus quitensis*; these contigs correspond to a genomic segment, termed RNA2, of novel viruses with two-component RNA genomes provisionally assigned to the Tecimovirids group related to the family *Benyviridae* [16]. Sequence analysis indicates that the second TCMB gene encodes a helicase domain-containing protein related to TGB1 and BMB1, whereas the proteins encoded by the third and fourth TCMB genes are similar to TGB2 and TGB3, respectively [14,16]. Interestingly, the protein encoded by the fourth TCMB gene also shows similarity to TGB2, suggesting that the 5′-distal gene in TGB and TCMB may have originated by a duplication of the TGB2/BMB2 gene of an ancestral BMB-like gene module [14]. Thus, TCMB represents a TGB-like gene module with an additional 5′-proximal gene, which is the characteristic feature of TCMB. This gene overlaps the downstream TGB1-like gene in both *D. scoparium* and *C. quitensis* and encodes a protein containing a double-stranded RNA (dsRNA)-binding domain of the DSRM_AtDRB family [16]. This protein has been given the name “vDRB” for viral dsRNA-binding protein. The DSRM_AtDRB domain of 65–70 amino acid residues in length is typical of a group of *Arabidopsis thaliana* dsRNA-binding proteins termed AtDRB1-AtDRB5, which contain two such domains, bind dsRNA and are involved in RNA-mediated silencing and dsRNA-mediated antiviral immunity [17,18]. The TCMB-encoded vDRB, unlike cell proteins, contains a single dsRNA-binding domain and a highly hydrophobic segment at the N-terminus [16]. To date, the vDRB protein has not been studied experimentally.

This paper demonstrates the ability of vDRB to bind RNA and shows that vDRB is a suppressor of RNA silencing in the context of viral infection but not in non-viral assays.

## 2. Results

### 2.1. RNA-Binding Properties of vDRB

**Figure 1:**
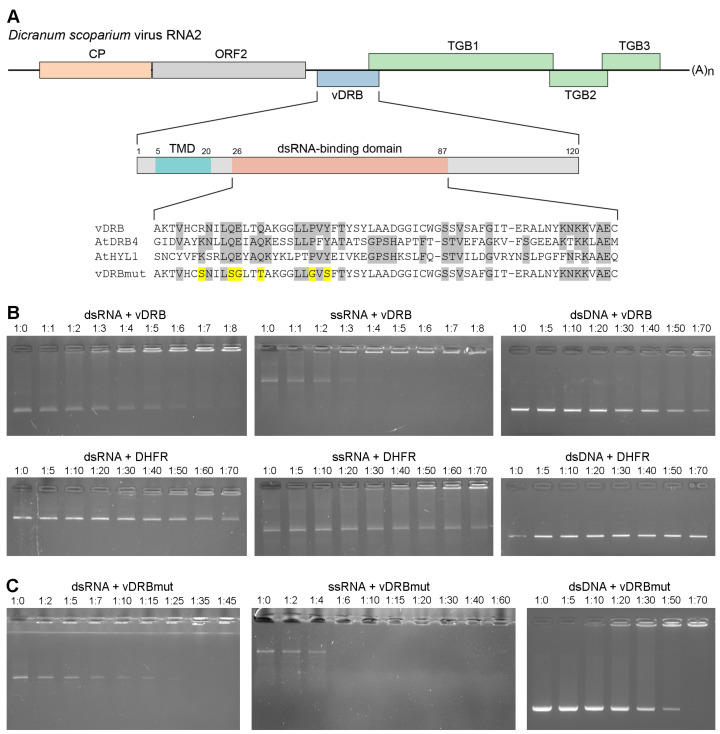
Analysis of the ability of vDRB to bind nucleic acids in gel-shift experiments. (**A**) Schematic representation of the genomic structure of *D. scoparium* virus RNA2 and molecular organization of vDRB. Proteins are shown as boxes. In vDRB, the positions of the transmembrane domain (TMD) and the dsRNA-binding domain are indicated by colored boxes. Numbers indicate the positions of the regions shown. Below the schematic, an alignment of the vDRB dsRNA-binding domain sequence with those of AtDRB4 and AtHYL1 is shown; gray shading indicates conserved amino acid residues. For vDRBmut, the introduced mutations are shown in yellow. (**B**) Binding of vDRB to dsRNA, ssRNA and dsDNA. In all panels, nucleic acids, as indicated above the gel images, were incubated with increasing amounts of either vDRB or DHFR (a negative control) and loaded onto the agarose gel. RNA:protein molar ratios are indicated above each lane. (**C**) Binding of vDRBmut to dsRNA, ssRNA and dsDNA. Nucleic acids were incubated with increasing amounts of vDRBmut and loaded onto the agarose gel. RNA:protein molar ratios are indicated above each lane.

Based on sequence analysis, the ORF3 product of *D. scoparium* virus RNA2 has been earlier termed viral DRB-like protein, or vDRB [16]; however, its ability to interact with RNA has not been tested so far. To verify whether vDRB can, indeed, bind dsRNA, the ORF3 product expressed in *Escherichia coli* was used. As the vDRB protein has an N-terminal hydrophobic sequence region (Figure 1A) suggested to be a transmembrane domain (TMD) [16], which is likely to be incompatible with a high-level expression in bacteria, a truncated version on the ORF3 gene encoding vDRB without the TMD was cloned into an expression vector as a fusion with a six-histidine-tag coding sequence. The recombinant vDRB protein was expressed in *E. coli* cells, affinity purified and renatured by dialysis. The ability of the resulting vDRB to bind nucleic acids was analyzed by use of a gel-shift assay, in which nucleic acids were incubated with increasing amounts of recombinant protein prior to analysis of samples in an ethidium bromide-containing non-denaturing agarose gel. As substrates for binding, GFP-specific single-stranded RNA (ssRNA), dsRNA and double-stranded DNA (dsDNA) were used.

The incubation of vDRB with dsRNA resulted in the formation of RNA-containing complexes unable to enter the gel. Such complexes first appeared at a protein:RNA molar ratio of 2:1, and at an 8:1 ratio the majority of input dsRNA was found in retarded complexes (Figure 1B). In a control gel-shift experiment, when *E. coli*-expressed purified mouse dihydrofolate reductase (DHFR), the protein lacking RNA-binding activity, was used for incubation with dsRNA, complexes that were unable to enter the gel, likely formed because of residual bacterial proteins non-specifically co-purified with DHFR, were first visible at a protein:RNA molar ratio of 30:1, and a significant amount of dsRNA remained unbound, even at a ratio of 70:1 (Figure 1B). These data demonstrate that vDRB is able to efficiently bind dsRNA and confirm that the observed binding is not due to bacterial proteins co-purified with vDRB. The incubation of vDRB with ssRNA revealed retarded complexes at a protein:RNA ratio of 1:1, whereas at a 5:1 ratio, all ssRNA was found in complexes unable to enter the gel (Figure 1B). Similar to dsRNA, the preparation of DHFR demonstrated much weaker ssRNA binding compared to vDRB (Figure 1A). Therefore, in addition to dsRNA binding, vDRB exhibits ssRNA-binding activity, which is comparable or even higher than the protein dsRNA-binding activity. In addition, vDRB demonstrated the formation of retarded complexes with DNA at high protein:DNA ratios, whereas no such complexes were found for DHFR under similar conditions (Figure 1B). These observations may reflect the presence of a weak non-specific affinity of vDRB to DNA.

Next, the role of the vDRB dsRNA-binding domain in dsRNA and ssRNA binding was investigated. Substitutions of conserved amino acid residues of the dsRNA-binding domain were introduced into the vDRB protein by site-directed mutagenesis (Figure 1A). The mutant, termed vDRBmut, was expressed in bacteria, purified and used for gel-shift analysis. In these experiments, vDRBmut exhibited considerably reduced ability for dsRNA binding. Indeed, in the presence of vDRBmut, the input dsRNA was fully incorporated into complexes unable to enter the gel at a protein:RNA ratio of 35:1 (Figure 1C), whereas vDRB caused a similar effect at an 8:1 ratio (Figure 1A). On the other hand, the introduced mutations were found to have little effect on ssRNA binding, as the input RNA was fully incorporated in the retarded complexes at a protein:RNA ratio of 6:1 in the case of vDRBmut (Figure 1C) and 5:1 in the case of vDRB (Figure 1B). These data demonstrate that the binding of dsRNA, but not ssRNA, is specified by the vDRB dsRNA-binding domain. Interestingly, vDRBmut exhibited increased dsDNA binding compared to vDRB (Figure 1B,C), suggesting that the introduced mutations may have resulted in an altered protein conformation favorable for non-specific interaction with dsDNA.

**Figure 2:**
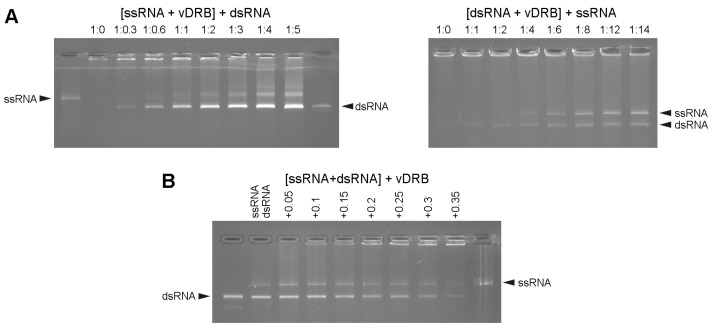
Analysis of the relative affinities of vDRB for dsRNA and ssRNA. (**A**) Analysis of the displacement of RNA bound by vDRB. Left, ssRNA was incubated with vDRB at a molar ratio of 1:4, when the majority of input ssRNA was incorporated into retarded complexes, then dsRNA was incubated with aliquots of the reaction mixture at the protein:RNA ratios indicated above the gel, and samples were loaded onto the gel. Right, dsRNA was incubated with vDRB at a ratio of 1:8 to form retarded complexes, then ssRNA was incubated with aliquots of the reaction mixture at the protein:RNA ratios indicated above the gel, and samples were loaded onto the gel. (**B**) Competition between dsRNA and ssRNA for binding to vDRB. A mixture of dsRNA and ssRNA was incubated with increasing amounts of vDRB as indicated above the gel in μg.

To estimate the relative affinities of vDRB to dsRNA and ssRNA, the ability of dsRNA to displace ssRNA from the complex with vDRB was analyzed. The vDRB was first incubated with ssRNA at a protein:RNA ratio of 4:1 that resulted in ssRNA incorporation into retarded complexes, then dsRNA was added to aliquots of the reaction in increasing concentrations. At dsRNA:protein ratios of 1:1 and higher, ssRNA was found to be partially displaced from complexes with vDRB, with increasing amounts of ssRNA being displaced as increasing amounts of dsRNA were added (Figure 2A). In a reciprocal experiment, when ssRNA was incubated with preformed complexes of dsRNA with vDRB, gradual displacement of dsRNA from the complexes was observed with increasing amounts of added ssRNA (Figure 2A). In addition, a competition experiment was carried out, in which vDRB was incubated with a mixture of ssRNA and dsRNA. As increasing amounts of vDRB were added, increasing amounts of both RNA types were incorporated into the retarded complexes, as evidenced by the amount of RNA that remained unbound (Figure 2B). Under these conditions, neither ssRNA nor dsRNA was preferentially bound by vDRB (Figure 2B). Taken together, the displacement and competition data show that vDRB binds dsRNA and ssRNA with similar affinities.

### 2.2. Subcellular Localization of vDRB

**Figure 3:**
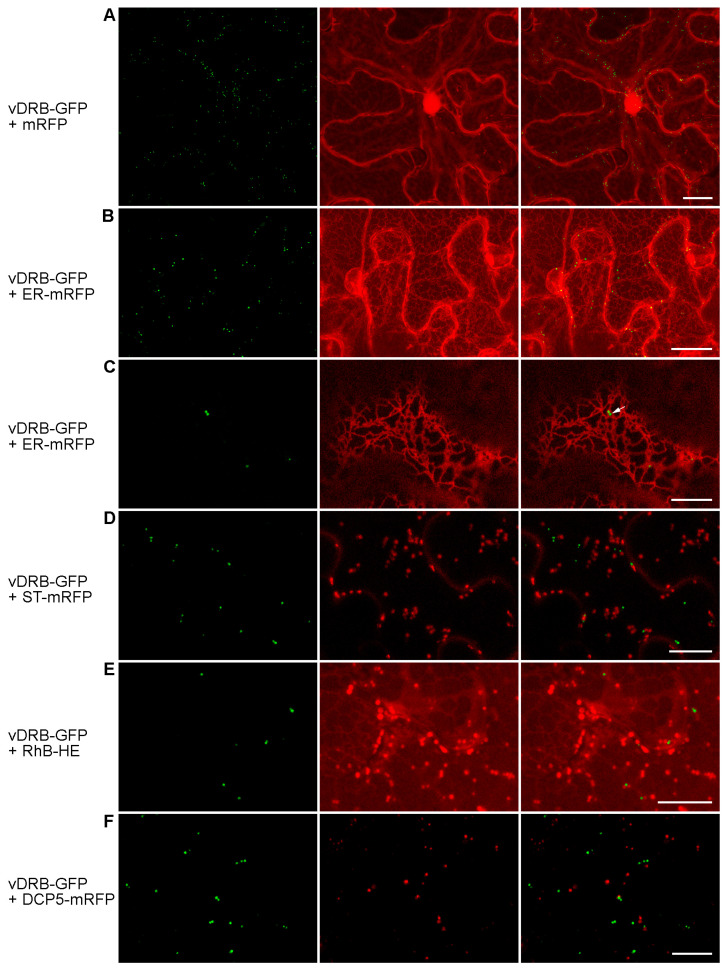
Subcellular localization of vDRB-GFP. vDRB-GFP was co-expressed with mRFP used to visualize the cell shape and the nuclei (**A**) or with markers of subcellular structures (**B**–**D**,**F**), as indicated on the left. In (**E**), vDRB-GFP-expressing leaf samples were stained with hexyl ester of rhodamine B. Left images, GFP channel. Center images, mRFP channel. Right images, superposition of images for GFP and mRFP channels. All images except C are reconstructed from Z series of optical sections. Images in C represent a single optical section. Scale bars, 20 μm (**A**,**B**) and 10 μm (**C**–**F**).

To analyze the subcellular localization of vDRB, the ORF3 coding region was fused to the GFP gene sequence to give the vDRB-GFP fusion gene, which was cloned in a binary vector under the control of the cauliflower mosaic virus (CaMV) 35S promoter. Agrobacteria carrying the expression cassette with the vDRB-GFP gene were used for the infiltration of *Nicotiana benthamiana* leaves. Confocal laser scanning microscopy of the agroinfiltrated leaves carried out 3 days post-infiltration (dpi) revealed that the vDRB-GFP protein was localized to numerous small bodies dispersed in the cytoplasm (Figure 3A).

To align the observed vDRB-GFP-containing structures with cell endomembranes, the fusion protein was co-expressed with one of two fluorescent markers, either the mRFP targeted to the ER lumen (ER-mRFP) or mRFP carrying the rat sialyltransferase (ST) signal peptide and located in the Golgi stacks (ST-mRFP). Confocal microscopy of agroinfiltrated *N. benthamiana* leaves revealed that the vDRB-specific bodies are associated with the tubules of the cortical ER (Figure 3B). However, no discrete structures corresponding to vDRB-GFP-containing bodies were visible in the mRFP channel (Figure 3B), and some vDRB-specific bodies, despite localization in close proximity to the ER membranes, did not contain ER-mRFP (Figure 3C). These observations indicate that these structures are not part of or directly derived from the ER. Co-expression with ST-mRFP revealed that the vDRB-specific bodies did not overlap with the Golgi stacks (Figure 3D). Therefore, experiments on co-expression of vDRB-GFP with ER-mRFP and ST-mRFP show that the vDRB-containing bodies are unrelated to the ER/Golgi-based secretion pathway.

To determine whether vDRB-specific bodies could be related to other elements of the cell endomembrane system, leaves agroinfiltrated for vDRB-GFP expression were treated with hexyl ester of rhodamine B, a cell-permeable dye that specifically stains membrane structures [19]. As revealed by confocal microscopy, the stain was found in numerous membranous structures in the cytoplasm; however, none of these structures overlapped with the vDRB-containing bodies (Figure 3E). Further, *N. benthamiana* leaves were agroinfiltrated for co-expression of vDRB-GFP with DCP5-mRFP, a marker of proteinaceous processing bodies, or P-bodies, involved in the translational repression and decay of mRNAs [20]. The GFP and mRFP signals did not overlap in examined cells (Figure 3F), showing that the vDRB-containing structures are unrelated to the P-bodies. Taken together, the data on vDRB-GFP co-localization with markers of cellular substructures demonstrate protein-specific localization but leave open the question of the origin and nature of the vDRB-containing cytoplasmic structures.

### 2.3. Suppression of RNA Silencing by vDRB in Non-Viral Experimental Systems

As vDRB was found to bind dsRNA in vitro, we further investigated whether this protein could disrupt the dsRNA-dependent pathways of RNA silencing and serve as a VSR. For these experiments, the vDRB coding sequence was cloned in a binary vector under the control of the CaMV 35S promoter. An agrobacterial culture carrying the resulting construct was used to identify the potential VSR activity of vDRB.

**Figure 4:**
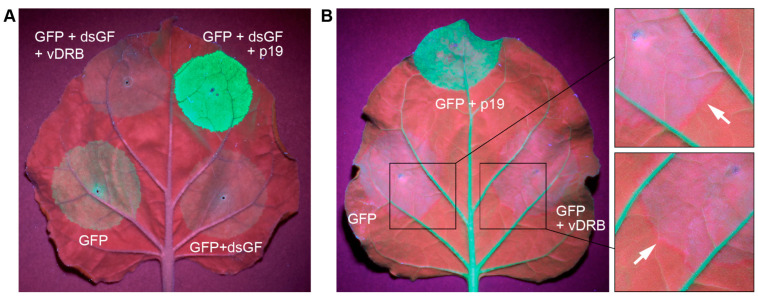
Analysis of the ability of vDRB to suppress RNA silencing in patch infiltration assays. (**A**) Analysis of the ability of vDRB to suppress dsRNA-induced silencing. *N. benthamiana* leaves were agroinfiltrated for expression of GFP, co-expression of GFP with dsGF (an inverted repeat construct that induces GFP-specific silencing), co-expression of GFP, dsGF and TBSV p19 (a well-characterized VSR) or co-expression of GFP, dsGF and vDRB. (**B**) Analysis of the potential of vDRB to affect cell-to-cell transport of silencing signals. Leaves of GFP-expressing transgenic *N. benthamiana* plants (line 16c) were agroinfiltrated for expression of GFP or co-infiltrated for co-expression of GFP with either vDRB or p19. Arrows in magnified leaf regions indicate red borders surrounding infiltrated areas. Leaves were imaged under UV light at 4 dpi (**A**) and 5 dpi (**B**).

First, the potential VSR activity was tested in a patch agroinfiltration assay involving the induction of RNA silencing by dsRNA [21]. In these experiments, *N. benthamiana* leaves were agroinfiltrated for (1) the expression of GFP, (2) co-expression of GFP with dsGF, an inverted repeat-containing transcript that forms a long dsRNA hairpin, which corresponds to two-thirds of the GFP sequence and acts as an inducer of GFP-specific silencing [21], (3) co-expression of GFP, dsGF and vDRB and (4) co-expression of GFP, dsGF and the tomato bushy stunt virus p19 protein, the well-characterized VSR [22,23], used as a positive control. Agroinfiltrated leaves were examined under UV light at 4 dpi. As expected, leaf areas agroinfiltrated for the expression of GFP produced a moderate level of GFP fluorescence, co-expression of GFP and dsGF resulted in no GFP signal due to GFP-specific silencing, whereas the co-expression of GFP, dsGF and p19 gave rise to an intense fluorescence in the agroinfiltrated area (Figure 4A). In contrast to p19, vDRB co-expressed with GFP and dsGF was unable to increase the level of GFP fluorescence compared to areas agroinfiltrated for the co-expression of GFP and dsGF (Figure 4A). Thus, vDRB was unable to suppress dsRNA-induced silencing in this experimental system.

Second, the ability of vDRB to affect the short-range (cell-to-cell) spread of silencing signals was examined using the infiltration of GFP-expressing transgenic *N. benthamiana* plants (16c line) [24], with agrobacteria carrying the GFP gene, which activates the GFP silencing in infiltrated areas. In this experimental setup, the transport of silencing signals can be visualized as a thin red border, 10–15 cells wide, surrounding the infiltrated leaf patches, resulting from the silencing of the GFP transgene in cells of the non-infiltrated area to which the silencing signal is transported from the infiltrated patches [25]. As expected, the infiltration of 16c plants with GFP-carrying agrobacteria resulted in the appearance of a red border around the infiltrated patches, whereas the co-infiltration of agrobacterial cultures carrying p19 and GFP genes, while greatly enhancing the GFP fluorescence, resulted in no visible red border (Figure 4B), as p19 is known to block the cell-to-cell transport of silencing signals [25]. Agrobacteria-mediated co-expression of GFP and vDRB in 16c plants resulted in a red border surrounding the infiltrated areas similar to that observed for GFP transient expression (Figure 4B). These observations show that vDRB does not affect the short-range transport of silencing signals in *N. benthamiana* leaves.

### 2.4. Suppression of RNA Silencing by vDRB in TCV-Based Assay

The potential of vDRB to suppress silencing was further analyzed in an assay using a modified genome of turnip crinkle virus (TCV) carrying the GFP gene replacing the gene for the TCV capsid protein, the viral VSR. The TCV-GFP construct, in the absence of VSR, is restricted to single initially infected leaf cells, whereas the co-expression of TCV-GFP with homologous or heterologous VSRs restores the viral ability for cell-to-cell transport, resulting in the formation of multicellular infection foci [26,27]. Therefore, as a control, halves of *N. benthamiana* leaves were infiltrated with a TCV-GFP-carrying agrobacterial culture, which was highly diluted to induce TCV-GFP expression in single cells that were distant from each other in the infiltrated area. The other halves of the leaves was co-agroinfiltrated with the highly diluted TCV-GFP culture and a vDRB-bearing culture used at normal dilution to enable vDRB expression in all cells of the infiltrated patches. Leaves were examined by epifluorescence microscopy at 5 dpi, and the number of cells comprising each individual TCV-GFP infection locus was recorded.

**Figure 5:**
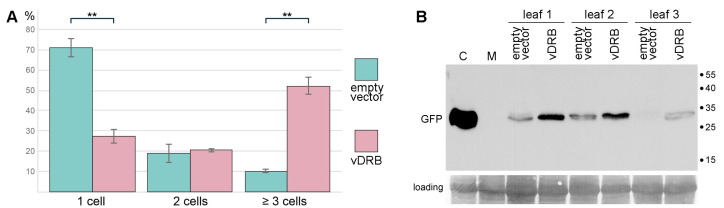
vDRB suppresses RNA silencing in TCV-based assay. (**A**) Percentage of fluorescent foci consisting of one, two and three and more cells observed upon co-expression of TCV-GFP with either vDRB or an empty vector. The number of foci observed on leaves of three plants (n) was as follows: vDRB, 241; vector, 183. Asterisks indicate statistically significant (** *p* < 0.01) differences according to the paired two-tailed Student’s *t*-test. The error bars indicate the standard error (SE). (**B**) Western blot analysis of three individual *N. benthamiana* leaves, one half of which was agroinfiltrated for co-expression of TCV-GFP and vDRB, and the other half for co-expression of TCV-GFP and empty vector. Samples were collected at 5 dpi. GFP-specific antibodies were used. The positions of molecular weight markers are shown on the right. M, mock sample (non-infiltrated leaf). C, control sample from GFP-expressing transgenic plants (line 16c). Membrane staining with Amido Black is shown below the Western blot to demonstrate gel loading.

Single-cell loci accounted for 70.8% in areas infiltrated for the expression of TCV-GFP alone and 27.3% in areas co-infiltrated for the expression of TCV-GFP and vDRB, showing a statistically significant difference (Figure 5A). Conversely, the percentage of loci consisting of three or more cells was significantly higher in the presence of vDRB (52.2%) compared to the control (10.2%). It should be noted that loci consisting of 10 or more cells were occasionally observed in leaf areas infiltrated for the co-expression of TCV-GFP and vDRB and were never found for TCV-GFP alone. These data demonstrate that vDRB enables the transport of TCV-GFP from cell to cell and significantly increases the size of TCV-GFP loci. To confirm these observations, samples of agroinfiltrated leaves were analyzed by Western blotting using GFP-specific antibodies. The amount of GFP was found to be considerably higher in leaves co-expressing TCV-GFP and vDRB compared to leaves expressing TCV-GFP alone (Figure 5B). Collectively, these data show that vDRB exhibits the characteristics of VSR in the TCV-based assay.

### 2.5. Influence of vDRB on PVX Infection

**Figure 6:**
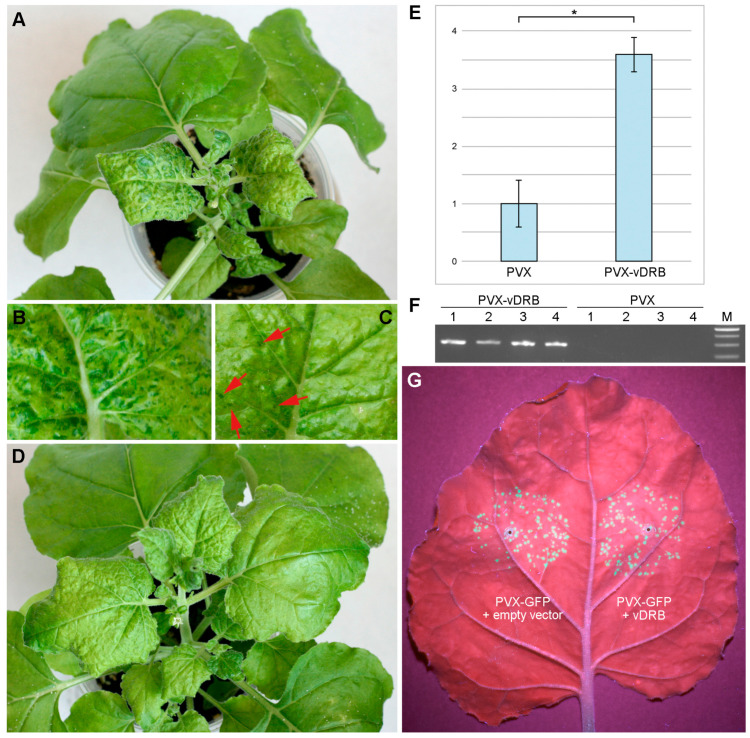
Effect of vDRB on PVX infection. Symptoms of virus infection in plants inoculated with PVX (**A**,**B**) and PVX-vDRB (**C**,**D**). The upper systemically infected leaves were imaged at 14 dpi. Images of magnified portions of upper leaves show the difference in number and size of DGIs induced by PVX (**B**) and PVX-vDRB (**C**). Red arrows indicate some DGIs on leaf infected with PVX-vDRB. (**E**) Virus accumulation in the upper leaves of plants infected with PVX and PVX-vDRB. Levels of virus genomic RNA were determined by quantitative reverse-transcription PCR (qPCR). The qPCR values were normalized to the internal control, RNA of an F-box protein. Data are mean from four biological replicates (n = 4). An asterisk denotes a statistically significant (*p* < 0.05) difference according to the paired two-tailed Student’s *t*-test. The error bars indicate the standard error (SE). (**F**) Reverse-transcription PCR detection of vDRB sequence in PVX-vDRB virus progeny at 14 dpi. PCR was carried out with vDRB-specific primers. Numbers indicate individual plants inoculated with either PVX or PVX-vDRB, as indicated. M, DNA size markers. (**G**) Analysis of the potential of vDRB to affect cell-to-cell transport of PVX. Leaf of *N. benthamiana* plants agroinfiltrated for co-expression of PVX with either vDRB or an empty vector, as indicated in the image. The leaf was imaged under UV light at 4 dpi.

VSRs have been shown to affect the symptoms of potato virus X (PVX) infection [28,29]. Therefore, vDRB was further evaluated for its ability to induce a similar effect. To this end, the vDRB coding sequence was cloned in a PVX-based vector PVX201, which is an infectious cDNA copy of the PVX genome modified for cloning and expression of foreign genes in PVX-infected cells. Leaves of young *N. benthamiana* plants were inoculated with either PVX-vDRB or the PVX vector construct used as a control. Typical symptoms of PVX infection, such as mosaic and distortion of upper plant leaves, first appeared in PVX-inoculated plants at 7 dpi, and all PVX-inoculated plants exhibited clearly visible symptoms at 8 dpi. In the case of plants inoculated with PVX-vDRB, there was a delay in the development of virus infection, as the symptoms first appeared in some inoculated plants at 8 dpi and were manifested in all plants at 10 dpi. The infection symptoms caused by PVX and PVX-vDRB at 10 dpi and later time points differed considerably. PVX-vDRB-induced symptoms were generally less severe than those caused by PVX; in particular, the upper leaves of PVX-vDRB-infected plants were less distorted and had much fewer ‘dark green islands’ (DGIs), which were also smaller than those of PVX-infected plants (Figure 6A–D). DGIs are areas of green leaf tissue that visually contrast with the surrounding chlorotic regions typical of virus infection; DGIs contain much less virus than chlorotic tissue and develop due to antiviral RNA silencing, leading to virus resistance in these local areas [30,31]. Therefore, the expression of vDRB in the context of PVX infection results in the inhibition of RNA silencing-dependent processes, leading to diminished formation of DGIs in virus-infected tissues, consistent with the VSR function of vDRB identified in the TCV-based experimental system. Analysis of the upper leaves by quantitative reverse-transcription-PCR at 14 dpi revealed a statistically significant difference in virus accumulation in plants inoculated with PVX and PVX-vDRB, as the level of PVX-vDRB RNA was 3.6-times higher than that of PVX (Figure 6E). As revealed by reverse-transcription-PCR, the vDRB sequence was maintained in PVX-vDRB virus progeny at 14 dpi (Figure 6F), suggesting that the effects observed in PVX-vDRB-infected plants result from the vDRB expression. As increased virus accumulation could result from enhanced virus transport in plants, the potential ability of vDRB to affect the cell-to-cell transport of PVX was investigated. The infectious PVX construct carrying the GFP gene (PVX-GFP) was used in these experiments. A PVX-GFP agrobacterial culture, highly diluted to initiate virus infection in single distant cells, was used to infiltrate *N. benthamiana* leaves in a mixture with either a culture for expression of vDRB or a culture carrying an empty expression vector. Agroinfiltrated leaves were examined under UV light at 4 dpi. No visible difference was found in the size of PVX infection loci in the presence and absence of vDRB (Figure 6G), indicating that the observed vDRB effect on PVX accumulation is not caused by enhanced virus cell-to-cell transport. Thus, the data on the influence of vDRB on PVX infection in *N. benthamiana* are consistent with the conclusion that virus accumulation in PVX-infected plants is significantly inhibited by an antiviral RNA-silencing response, while vDRB expressed in the context of PVX infection can suppress antiviral silencing and, thus, increase virus accumulation and alter the phenotype of induced symptoms.

## 3. Discussion

The hallmark of TCMB is the 5′-proximal gene coding for vDRB, a small protein that contains a region closely related to the dsRNA-binding domain found in a group of cell DRB proteins, including DRB4 and HYL1 [16]. In the gel-shift experiments reported here, vDRB is shown to efficiently bind to both dsRNA and ssRNA but not dsDNA, which is only partially bound by vDRB at high protein:RNA ratios. Whereas the dsRNA binding was anticipated, the ssRNA binding, which appears to be as efficient as the interaction with dsRNA, was not predicted. Similar to vDRB, AtHYL1 has been shown to interact with ssRNA, while AtDRB4 has no RNA-binding ability [32]. A possible clue to unraveling the vDRB ssRNA-binding activity may be found in experiments with vDRBmut, a mutated version of vDRB with substitutions of key amino acid residues of the dsRNA-binding domain. The mutations introduced in vDRB decrease dsRNA binding by approximately five-fold without a pronounced effect on ssRNA binding. These observations suggest that ssRNA binding does not involve the conserved residues of the dsRNA-binding domain and, therefore, is likely independent of dsRNA binding. This additional binding activity may be hypothesized to result from non-specific electrostatic interactions with negatively charged nucleic acids. Such interaction can account for the decreased, compared to the wildtype protein, but detectable interaction of vDRBmut to dsRNA and weak interaction of vDRB with dsDNA. Interestingly, vDRBmut appeared to show, likely due to altered conformation of the mutant protein, an increased ability for dsDNA binding, supporting the hypothesis that non-specific binding of vDRB to nucleic acids does not depend on the ability to interact with dsRNA.

The TCV-based assay indicates that the vDRB protein suppresses RNA silencing. The VSR activity of vDRB is consistent with its effect on PVX infection. In fact, the expression of vDRB in the context of the PVX genome causes a marked reduction in both the number and size of DGIs, which are regions of infected leaves where virus accumulation is reduced by locally activated RNA silencing [30,31]. The symptoms of PVX-vDRB infection are less severe than those of PVX, whereas the expression of other VSRs leads to more severe PVX symptoms and the systemic necrosis of infected plants [28,29]. This effect has been attributed to the VSR-induced increase in the level of PVX TGB1, which, when its amount reaches a certain threshold, triggers a hypersensitive response [33]. It should be noted that the expression of VSRs typically does not lead to an increase in the PVX RNA level [28,34,35,36], whereas vDRB induces a 3.6-fold increase in PVX RNA accumulation. Currently, it remains to be studied why the vDRB-induced increased virus accumulation, which potentially leads to higher TGB1 expression levels, does not result in systemic necrosis. We hypothesize that, in addition to its VSR function, vDRB can influence the mounting of other plant responses to virus infection that is manifested by milder, compared to PVX, symptoms induced by PVX-vDRB. Therefore, in its effect on PVX infection, vDRB differs from other VSRs in at least two aspects: (1) vDRB induces milder rather than more severe symptoms, and (2) vDRB induces an increase in virus accumulation. These observations indicate that the vDRB-specific mechanism of silencing suppression can differ from that of other VSRs analyzed for their effect on PVX infection.

The molecular details of the vDRB-specific VSR activity are a subject of further studies. In general, vDRB, being a dsRNA-binding protein, can suppress antiviral RNA silencing by interacting with either the viral dsRNA replication intermediate, as shown for P14 of pothos latent virus and TCV P38, or virus-specific double-stranded small-interfering RNA (siRNA), as demonstrated for tomato bushy stunt virus p19 [37]. Alternatively, vDRB may compete with one of DRB domain-containing proteins like DRB2, an antiviral effector [38], thus inhibiting silencing-based antiviral defense mechanisms. In addition, as viral dsRNA can activate a pattern-triggered immunity pathway that leads to enhanced callose deposition at PD, restricting therefore virus cell-to-cell transport [39], the inhibition of this antiviral defense pathway by vDRB may be presumed as a possible mechanism of vDRB influence on virus infection. However, as the expression of vDRB does not affect the cell-to-cell transport of PVX, this possibility seems unlikely. Considering the mechanism of silencing suppression by vDRB, it should be noted that the VSR function of vDRB is only manifested in the context of virus infection, either that of TCV or PVX tested in this study, and not in artificial systems such as non-viral transient expression assays, suggesting a link between the vDRB activity and virus replication. Similar to vDRB, the 29K MP of tobacco rattle virus (TRV) has been shown to exhibit the VSR properties only in the context of viral RNA replication but not in an agroinfiltration assay [40]; however, the mechanism of the replication-dependent silencing suppression remains unknown. Conceivably, the TRV MP and vDRB can suppress virus replication-targeting components of the RNA-silencing machinery, rather than those common for silencing of viral and non-viral RNAs.

Unlike cell DRB proteins, vDRB has an N-terminal hydrophobic region [16]. This region can function either as a signal peptide that cotranslationally guides the protein to the ER lumen and is cleaved off to give the mature protein or as an uncleavable membrane anchor that directs cotranslational integration into the ER membrane. In either case, the presence of the hydrophobic region implies vDRB localization to membrane structures. Unexpectedly, vDRB-GFP is found in small cytoplasmic bodies that do not contain markers of the ER and Golgi structures and, moreover, are not stained with a membrane-specific dye. We hypothesize that vDRB may be translocated, due to the cleavable N-terminal signal peptide, into the ER lumen and, then, in its mature form, retrotranslocated into the cytoplasm, avoiding degradation by the 26S proteasome complex. A similar translocation pathway leading to protein cytoplasmic localization has been shown for the ORF2 protein of hepatitis E virus [41] and the precore protein of hepatitis B virus [42]. Undoubtedly, this hypothesis requires experimental verification, and additional studies are needed to determine the identity of the structures to which vDRB localizes in plant cells.

While the dsRNA-binding domains of the DSRM_AtDRB family, such as that present in vDRB, have not been reported so far for other virus-encoded proteins, other types of dsRNA-binding domains are known to be encoded by genomes of certain RNA viruses. For example, sweet potato chlorotic stunt virus (SPCSV, a crinivirus) encodes a protein containing an RNAse III-like dsRNA-binding domain (cd10845) [43], whereas the B2 protein of flockhouse virus (FHV), which can infect both insect and plant cells, contains a dsRNA-binding domain of superfamily cl12995 [44]. The SPCSV RNAse III, being unable to function as VSR on its own, enhances the silencing suppressing activity of p22, the SPCSV VSR [45], playing, therefore, an accessory role that is not currently known. In contrast, the FHV B2 protein is a potent VSR [44,46] that acts by binding to dsRNA of various sizes, thereby protecting double-stranded virus replication intermediates from cleavage by dicing enzymes and inhibiting incorporation of double-stranded siRNA into effector complexes [44]. vDRB is unlikely to suppress silencing in a manner similar to that of FHV B2, as it is unable to suppress silencing induced by dsRNA in a patch agroinfiltration assay.

Taken together, the data presented in this paper suggest that vDRB is structurally and functionally unique among virus-encoded proteins. In particular, being a dsRNA-binding protein, vDRB, unlike other virus-encoded VSRs, is unable to suppress dsRNA-induced silencing in the agroinfiltration-based experimental system and exhibits VSR properties only in the context of virus infection. Therefore, vDRB, which has silencing suppression characteristics unusual for other viral VSRs, requires further investigation to elucidate the molecular mechanism of vDRB-specific RNA silencing suppression.

## 4. Materials and Methods

### 4.1. Recombinant Constructs

The recombinant constructs for the transient expression of mRFP, ER-mRFP and ST-mRFP in plants [13], as well as PVX-GFP [47], PVX201 [48], TCV-GFP [26,27] and pBSCII-SK(+)-GFP [49] were described previously. The construct DCP5-mRFP was kindly provided by Dr. Nina Lukhovitskaya (University of Cambridge). Primers used to generate other recombinant clones are listed in Supplementary Table S1.

The *D. scoparium* vDRB gene was chemically synthesized (Evrogen, Moscow, Russia) and subcloned into a binary vector pLH* [50] for expression in plants. For expression in *E. coli*, a sequence region encoding vDRB without the N-terminal hydrophobic domain was amplified with specific primers vDRB-pET-P and vDRB-pET-M. The resulting amplification product was digested with *Bam*HI and *Xho*I and cloned into pET-33b(+) (Novagen, Madison, WI, USA). To introduce mutations into vDRB, overlap-PCR with primers vDRB-ovl-P and vDRB-ovl-M was carried out. For DHFR production in *E. coli*, the expression vector pQE-40 (Qiagen, Hilden, Germany) was used. For expression of vDRB from the PVX vector, the vDRB-coding sequence was amplified with primers vDRB-201-P1 and vDRB-201-M. The resulting product was digested with *Nhe*I and *Sal*I and cloned into PVX201. All constructs were verified by sequencing.

### 4.2. Protein Expression in E. coli

*E. coli* cells (strain BL21) were transformed with expression vectors, and clones with the highest expression levels were selected. For recombinant protein expression, clones were grown overnight at 37 °C in 2YT medium in the presence of kanamycin (25 μg/mL). The overnight culture was diluted 10-fold and grown at 37 °C until an optical density at 600 nm (OD_600_)  =  0.8 was reached. Protein expression was induced by addition of IPTG to a final concentration of 1–2 mM and further culture growth for 2–4 h. Cells were pelleted at 4500 g for 10 min. The recombinant proteins carrying the N-terminal 6xHis tag were purified on Ni-NTA agarose (Qiagen) according to the manufacturer’s protocol. Purified proteins were analyzed by SDS electrophoresis in a 15% polyacrylamide gel according to Laemmli and renatured by dialysis.

### 4.3. Gel-Shift Experiments

RNA used in gel-shift experiments was obtained by in vitro transcription with T7 RNA polymerase (ThermoFisher Scientific, Waltham, MA, USA). The ssRNA substrate was obtained by transcription of the pBSCII-SK(+)-GFP linearized with *Xba*I. The dsGFP RNA substrate was synthesized by transcription of a PCR product obtained on the template of pBSCII-SK(+)-GFP with a pair of T7 promoter-containing primers dsC3-P and dsC3-M (Supplementary Table S1). The dsDNA substrate was obtained by PCR with universal and reverse sequencing primers on the template of pBSCII-SK(+)-GFP.

In gel-shift experiments, nucleic acids were incubated with variable amounts of a protein in a binding buffer (10 mM Tris-HCl pH 7.5, 100 mM NaCl, 5 mM ZnCl_2_) for 30 min on ice. The samples were analyzed in 1.5% non-denaturing agarose gel containing ethidium bromide. For displacement experiments, initial complex was formed in the binding buffer at protein:RNA ratios when the majority of RNA was bound for 30 min on ice; then, a displacing RNA was added to aliquots of initial complex at different protein:RNA ratios and incubated for 30 min on ice prior to loading onto an agarose gel.

### 4.4. Plant Material

*N. benthamiana* plants were grown under standard conditions (16 h/8 h light/dark cycles, 24/20 °C day/night temperatures, 50% humidity) in a glasshouse or in growth chambers. The 5- to 6-week-old plants were used for agroinfiltration. The 3-week-old plants were used for mechanical inoculation.

### 4.5. Plant Agroinfiltration and Inoculation

*Agrobacterium tumefaciens* (strain C58C1) cells were transformed with binary vectors using a freeze–thaw method. Prior to agroinfiltration, bacterial cultures were grown as described previously [50]. In brief, agrobacteria were grown in Luria-Bertani (LB) medium at 28 °C with appropriative antibiotics, 10 mm 2-(*N*-morpholino)ethanesulfonic acid (MES), pH 5.5 and 20 μm acetosyringone. Cells were collected by centrifugation, resuspended in infiltration buffer (10 mM MES, pH 5.5, 10 mM MgCl_2_, 150 mM acetosyringone) and incubated at room temperature for 3 h. For infiltration, *A. tumefaciens* suspensions were diluted to a final OD_600_ = 0.3. For analysis of PVX-GFP cell-to-cell transport, the PVX-GFP agrobacterial culture carrying the PVX-GFP construct was infiltrated at OD_600_ = 0.00003. Similarly, for the TCV-based assay, the agrobacterial culture carrying the TCV-GFP was diluted to OD_600_ = 0.00024. The abaxial surface of *N. benthamiana* leaves was agroinfiltrated with a 2 mL needle-less syringe. For analysis of PVX and PVX-vDRB infection, leaves of *N. benthamiana* were dusted with carborundum and mechanically inoculated with plasmid DNA; 7 μg DNA (1 μg/μL) was used for inoculation of each leaf.

### 4.6. Western Blot Analysis

Samples *of N. benthamiana* leaves were ground to a powder in liquid nitrogen; then, a buffer containing three parts of Tris-HCl, pH 7.5 and one part of 4× Laemmli sample buffer (100 mM Tris-HCl pH 6.8, 100 mM β-mercaptoethanol, 10% glycerol, 4% SDS, 0.1% bromophenol blue) was added. The samples were incubated at 95 °C for 5 min, centrifugated to remove cell debris and loaded onto 12% polyacrylamide SDS-PAGE. After electrophoresis, proteins were transferred to a Hybond-P membrane (GE Healthcare Bio-Sciences, Niskayuna, NY, USA). Peroxidase-conjugated rabbit Anti-GFP antibodies (Rockland, Pottstown, PA, USA) and the ECL system (GE Healthcare Bio-Sciences) were used for protein detection.

### 4.7. Quantitative PCR

Leaf discs were frozen in liquid nitrogen and ground to fine powder. Total RNA was extracted using ExtractRNA reagent (Evrogen) according to manufacturer’s instructions. To avoid contamination with plant genomic DNA, samples were treated with RNase-free DNAseI (ThermoFisher Scientific). After DNase treatment, 1 µg of each RNA sample was transcribed into cDNA with oligo(dT) primer using a Revertaid reverse transcriptase (ThermoFisher Scientific). Then, 1 μL of 3-fold-diluted reverse-transcription product was used for real-time PCR reactions with a qPCRMix M-440 (Synthol, Moscow, Russia) using PVX RNA-specific primers PVX-REP-P and PVX-REP-M and primers for the reference mRNA of F-box protein, F-box-F and F-box-R (Supplementary Table S1). Reactions were carried out in the CFX Connect Real-Time PCR System (Bio-Rad, Hercules, CA, USA). The *C*_t_ value for PVX RNA was normalized to the reference gene mRNA.

### 4.8. Microscopy and Virus Movement Visualization

Analysis of protein subcellular localization was carried out using a confocal laser scanning microscope Nikon C2plus (Tokyo, Japan) equipped with a ×60 (1.2 NA) water immersion objective. Excitation wavelengths were 488 nm for GFP and 548 nm for mRFP and hexyl ester of rhodamine B. Acquisition bands were 495–545 nm for GFP and 580–640 nm for mRFP. Samples of agroinfiltrated leaves were analyzed using confocal microscopy at 3 dpi. Images were processed with Nikon NIS Elements software (version 5.21.00). The foci of TCV-GFP infection were examined using Zeiss Axiovert 200 M epifluorescent microscope (Zeiss, Oberkochen, Germany) with a ×20 objective. PVX-GFP infection loci were observed under long-wave UV light (365 nm) using a Black-Ray B-100AP lamp (UVP, Cambridge, UK).

## Data Availability

Not applicable.

## Supplementary Materials

The supporting information can be downloaded at: https://www.mdpi.com/article/10.3390/ijms241814144/s1.
