# Supplementary material for: Properties of Plant Virus Protein Encoded by the 5′-Proximal Gene of Tetra-Cistron Movement Block
Source: Int J Mol Sci. 2023 Sep 15;24(18):14144. doi: 10.3390/ijms241814144 (PMC10532019; doi:10.3390/ijms241814144)
Supplement: Supplementary file 1 [file ijms-24-14144-s001.zip › ijms-2572775-supplementary.pdf]

Supplementary Table S1. Primers used in this study.

Primer name	Sequence, 5' to 3'
vDRB-pET-P	GCGGATCCGGGCGGTAGTGAAGGTAGAGTTGCTAAAACT
vDRB-pET-M	CGCTCGAGTTAGGTGTCTCTTTTAGATAAGAGTTC
vDRB-ovl-P	CTGACAACAGCCAAGGGTGGGTTGTTGGGTGTCTCCTTTACG
vDRB-ovl-M	CACCCTTGGCTGTTGTCAGACCGCTCAAAATATTGCTACAATG
vDRB-201-P1	GGGCTAGCATGAGTAGGTCTTTTCTCGTTCTCGTTG
vDRB-201-M	CCCGTCGACTTAGGTGTCTCTTTTAGATAAGAGTT
dsC3-P	GAGAATTCTAATACGACTCACTATAGGGTGAAGGTGATGCTACATA
dsC3-M	GAGAATTCTAATACGACTCACTATAGGGCAGATTGTGTCGACAG
PVX-REP-P	CATTGTATGCGACCTTAGTTCTC
PVX-REP-M	CGTGTAATTGAGATGTCCGAGAA
F-box-F	GGCACTCACAAACGTCTATTTC
F-box-R	ACCTGGGAGGCATCCTGCTTAT
